# Two new *Russula* species (fungi) from dry dipterocarp forest in Thailand suggest niche specialization to this habitat type

**DOI:** 10.1038/s41598-022-06836-x

**Published:** 2022-02-18

**Authors:** Komsit Wisitrassameewong, Cathrin Manz, Felix Hampe, Brian P. Looney, Thitiya Boonpratuang, Annemieke Verbeken, Tuksaporn Thummarukcharoen, Tanakorn Apichitnaranon, Maneerat Pobkwamsuk, Miroslav Caboň, Slavomír Adamčík

**Affiliations:** 1 National Biobank of Thailand, National Science and Technology Development Agency (NSTDA), Thailand Science Park, Khlong Nueng, Khlong Luang, Pathum Thani 12120 Thailand; 2 Mycology Research Group, Faculty of Biological Sciences, Goethe University Frankfurt am Main, Max-von-Laue Straße 13, 60438 Frankfurt am Main, Germany; 3 Butzbach, Germany; 4 Department of Biology, Clark University, Worcester, MA USA; 5 Research Group Mycology, Department of Biology, Ghent University, K.L. Ledeganckstraat 35, 9000 Ghent, Belgium; 6 Plant Science and Biodiversity Centre, Slovak Academy of Sciences, Dúbravská cesta 9, 845 23 Bratislava, Slovakia

**Keywords:** Ecology, Evolution, Molecular biology, Plant sciences

## Abstract

Dry dipterocarp forests are among the most common habitat types in Thailand. Russulaceae are known as common ectomycorrhizal symbionts of Dipterocarpaceae trees in this type of habitat. The present study aims to identify collections of *Russula* subsection *Amoeninae* Buyck from dry dipterocarp forests in Thailand. A multi-locus phylogenetic analysis placed Thai *Amoeninae* collections in two novel lineages, and they are described here as *R. bellissima* sp. nov. and *R. luteonana* sp. nov. The closest identified relatives of both species were sequestrate species suggesting that they may belong to drought-adapted lineages. An analysis of publicly available ITS sequences in *R.* subsect. *Amoeninae* did not confirm evidence of any of the new species occurring in other Asian regions, indicating that dry dipterocarp forests might harbor a novel community of ectomycorrhizal fungi. Macromorphological characters are variable and are not totally reliable for distinguishing the new species from other previously described Asian *Amoeninae* species. Both new species are defined by a combination of differentiated micromorphological characteristics in spore ornamentation, hymenial cystidia and hyphal terminations in the pileipellis. The new *Amoeninae* species may correspond to some *Russula* species collected for consumption in Thailand, and the detailed description of the new species can be used for better identification of edible species and food safety in the region.

## Introduction

*Russula* Pers. is a genus of ectomycorrhizal agarics and truffles (Basidiomycota) distributed globally. It can be found in diverse forest ecosystems, ranging from the arctic to the tropics. *Russula* is currently classified into 8 subgenera: *Russula* subg. *Archaea* Buyck & V. Hofst., *R.* subg. *Brevipedum* Buyck & V. Hofst., *R.* subg. *Compactae* (Fr.) Bon, *R.* subg. *Crassotunicata* Buyck & V. Hofst., *R.* subg. *Glutinosae* Buyck & X.H. Wang, *R.* subg. *Heterophyllidiae* Romagn., *R.* subg. *Malodorae* Buyck & V. Hofst., and *R.* subg. *Russula*^1,2^. Among the most species diverse subgenera, *R.* subg. *Heterophyllidia* comprises species with diverse basidiocarp colour and microscopic features. Recent multilocus phylogenies have distinguished several well-defined lineages ranked as sections within this subgenus such as *R.* sect. *Aureotactae* Buyck & V. Hofst., *R.* subsect. *Cyanoxanthinae* Singer, *R.* sect. *Heterophyllae* Fr., *R.* sect. *Ingratae* Quél., *R.* subsect. *Ilicinae* Buyck., *R.* subsect. *Oleiferinae* Buyck and *R.* sect. *Subvelatae* Singer^1^.

The diversity of *Russula* in Thailand is estimated to be high and particularly well represented in broadleaf forests dominated by Dipterocarpaceae or Fagaceae trees^3,4^. Many edible *Russula* species are gathered in rural regions in northern and northeastern Thailand. However, local mushroom hunters are accustomed to recognizing edible russulas that represent morphological complexes and their identifications to species are unprecise. These field identifications rely on incomplete macromorphological descriptions and even local fungal inventories regularly apply names of American, European or Japanese origin for Thai species^5^. For example, names of the European taxa *R. emetica* (Schaeff.) Pers. and *R. violeipes* Quél. were often used for red-capped species^5^, while current studies have demonstrated that the distributions of European and American taxa are unlikely to extend to southeast Asia. The situation in Asia is more challenging. Some species are apparently distributed in larger areas and even separated by the sea, e.g. *R. castanopsidis* Hongo known from Japan and Korea^6^ or *R. bella* Hongo distributed in Japan, Korea and China^7^. Some earlier studies have included a number of Thai collections in ITS phylogenies, but these studies had insufficient sampling of closely related taxa to determine species limits^8–10^. Only a small number of species have been described as new and illustrated in detail from Thailand, and all these reports are relatively new, i.e. *R. siamensis* Yomyart, Piapukiew, Watling, Whalley & Sihan^11^, *R. aurantiopectinata* F. Hampe & Manz, *R. magica* Manz & F. Hampe^12^ and *R. purpureogracilis* F. Hampe, Looney & Manz^6^.

This study focuses on *Russula* diversity of dry dipterocarp forest. Forests dominated by members of the family Dipterocarpaceae are one of the most common and important ecosystems in tropical regions of Asia. They are distributed throughout the Southeast Asian realm, e.g. Malesia, the Mainland Southeast Asia, Indochina Peninsula, South Asia, Sri Lanka and Seychelles Islands^13^. The northernmost distribution of the family in the Indo-Malayan realm is located in subtropical southern China. The dipterocarp tree community of the Indochina Peninsula is specific and different from adjacent areas^14^. The endemicity of Dipterocarpaceae in Asia is relatively high at the extremes of their geographical distribution such as in Sri Lanka, South India, China mainland as well as Malesia, while it is very low in the centre of the Indochina peninsula^15^. Compositions of tree species in forests in northern, northeastern and eastern Thailand are relatively similar^16^. In Thailand, the family Dipterocarpaceae is represented by 8 genera and 63 species^17^ and is distributed in all parts of the country. The majority of species are found in either gallery forests, mixed evergreen or mixed deciduous forests. Only 5 species are xerophytic, which grow on sandy soil in the dry dipterocarp forests; *Dipterocarpus obtusiformis*, *D. tuberculatus*, *D. intricatus*, *Shorea obtusa*, and *S. siamensis*. Dry dipterocarp forests in the north and east of the country are widely distributed and frequent forest types along the Indochina Peninsula and are characterized by relatively low precipitation (annual rainfall of 1000–1500 mm), an open canopy and the presence of abundant large herbivores grazing the understory. Core areas of dry dipterocarp forests are located in Myanmar, Thailand, and Cambodia^18^. In northern Thailand, where the relief of the country topography is more rugged, dry dipterocarp forests occur at lower elevation^19^. In the eastern part of the country they also occur in lowlands and low elevations but their occurrence is even more fragmented probably due to intensive human land use^9,20^.

This study aims to estimate the diversity of *R.* subsection *Amoeninae* in dry dipterocarp forests of north and northeast Thailand based on recently collected materials. This subsection is classified in *R.* sect. *Heterophyllae* and is morphologically characterised by spores with a subreticulate or reticulate ornamentation, the absence of gloeopherous cystidia in the hymenium and pileipellis, and the presence of subulate hyphal terminations both in the pileipellis and on the lamellae edges^21^. *Russula* subsect. *Amoeninae* is a well-defined phylogenetic lineage, and, based on publicly available ITS sequence data, it includes at least 30 species worldwide, eight of which are Asian and five have been formally described^7^. In this study, we will specify whether species of *Amoeninae* found in Thailand match any of the previously described members of the lineage or whether they represent new species.

## Results

### Phylogenetic analyses

**Figure 1:**
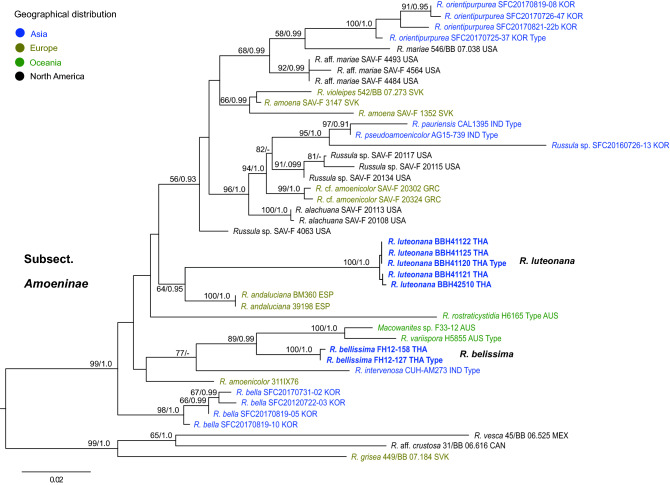
ML phylogenetic tree inferred from the three-gene dataset (ITS, *rpb2*, mtSSU) of *Russula* subsection *Amoeninae* species, using ML and BI analyses. Three members of *R.* subg. *Heterophyllidiae* are used as outgroup. Species in boldface are new species in this study. Bootstrap support values (BS ≥ 50%) and posterior probabilities (PP ≥ 0.90) are shown at the supported branches.

A total of 21 sequences were newly generated and deposited in GenBank (Supplementary Table 1). The concatenated sequence alignment of the three loci comprised 100 sequences (38 for ITS, 30 for *rpb2* and 32 for mtSSU) from 43 collections (Supplementary Table 1). The alignment was 2,004 characters long, including gaps. Multi-locus trees generated from ML and BI analyses showed similar topologies without any supported topological conflict. The multi-locus phylogeny (Fig. 1) confirmed placement of all Thai collections within the well-supported *R.* subsect. *Amoeninae* (ML = 99, BI = 1.0). Five collections from northeastern Thailand and two collections from northern Thailand form two strongly supported clades and are described below as the new species *R. bellissima* sp. nov. and *R. luteonana* sp. nov. The new species are not resolved as sister. The first species, *R. bellissima,* is strongly supported as sister to a clade of Australian sequestrate species that includes *R. variispora* T. Lebel and an undescribed *Russula* sp. labeled as *Macowanites* sp. The Indian species *R. intervenosa* S. Paloi, A.K. Dutta & K. Acharya is placed as sister to them with bootstrap support of 77. The second species, *R. luteonana*, is placed with moderate support as sister to the sequestrate European species *R. andaluciana* T.F. Elliott & Trappe.

**Figure 2:**
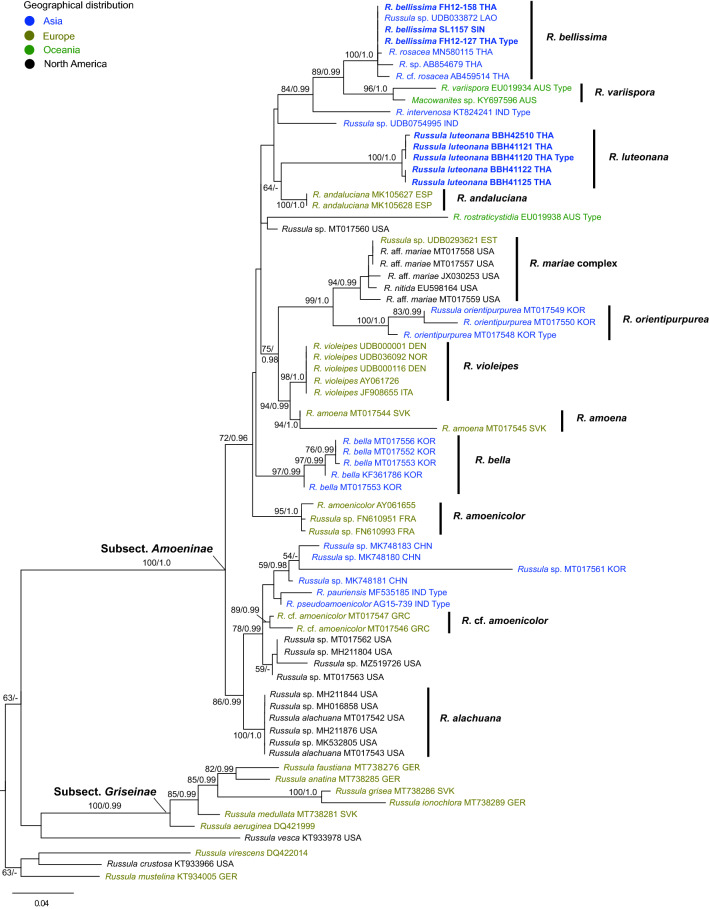
ML phylogenetic tree inferred from the ITS region of *Russula* subsection *Amoeninae* species and allied groups, using ML and BI methods. Samples in boldface are new species in this study. Bootstrap support values (BS ≥ 50%) and posterior probabilities (PP ≥ 0.90) are shown at the supported branches.

The ITS tree (Fig. 2) shows a similar topology and relationships for the studied specimens. In addition, *R. intervenosa* received good support (ML = 84, BI = 0.99) as sister to the clade of *R. bellissima* and *R. variispora*. Five additional ITS sequences that are grouped with strong support within *R. bellissima* species clade were recovered, three from Thailand, one from Laos, and one from Singapore. We did not recover any other *Amoeninae* ITS sequences from Thailand.

### Taxonomy

#### *Russula bellissima* Manz & F. Hampe sp. nov.

##### *Mycobank*: MB 840549

*Holotype* THAILAND, Theong district, Chiang Rai, 19°36′45''N 100°4′00''E, alt. 500 m, dry dipterocarpus forest in small groups on loamy soil, 12 July 2012, F. Hampe (Holotype: GENT FH 12-127; Isotype: MFLU12-0619).

*Etymology ’bellus’* = latin for beautiful, pretty, lovely; *’bellissima’* = the most beautiful. Resembling the species *Russula bella* which is also belonging to *Russula* subsection *Amoeninae.*

*Diagnosis* Pileus small to medium-sized; cuticle dry, smooth, matt and pruinose, red; stipe white or with a red flush; spore ornamentation of moderately distant to dense amyloid spines or warts, frequently fused into short crests or even long wings; suprahilar spot inamyloid; hymenial cystidia and pileocystidia absent.

**Figure 3:**
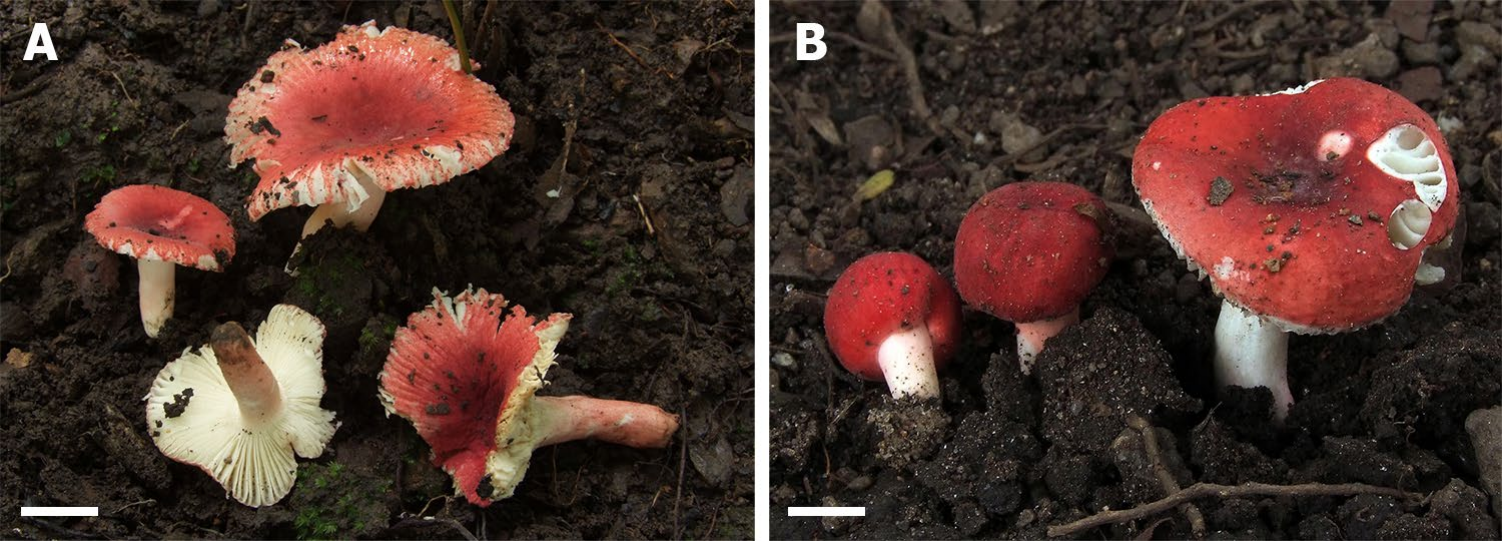
Basidiomata of *Russula bellissima*. (**A**) FH12-127 (Holotype). (**B**) FH12-158. Scale bar = 1 cm. Photos by Felix Hampe.

*Pileus* (Fig. 3) small to medium sized, 10–50 mm diam., young hemispherical or convex, becoming plane and depressed at the centre; margin first even, when old distinctly tuberculate-striate up to 10 mm from the margin, often radially cracking; cuticle hardly peeling, radially disrupted into small patches, pruinose when young, later dry, smooth, matt and pruinose in the centre, colour near the margin when young varnish red (9C8), later red to coral red (9B6-7); near the centre deep red, blood red, dark red (10C7-8), raspberry red (10D7), strawberry red (10D8) or purple brown (10E-F8). *Lamellae*: 3–5 mm deep, thin, moderately dense, 6–8 at 1 cm near the pileus margin, adnexed, white, slightly anastomosing at the base; lamellulae absent, occasionally forked near the stipe; edges concolorous, entire but pruinose under lens. *Stipe*: 10–30 × 3–7 mm, usually narrowed towards the base, sometimes cylindrical, surface smooth, white and mainly with a distinct pastel red to red flush, occasionally completely white or sometimes also almost completely red, interior stuffed. *Context*: white, fragile, unchanging when damaged, reaction with guaiac after 5 s negative on both stipe and lamellae surfaces, reaction to FeSO_4_ and sulfovanillin negative; taste mild; odour inconspicuous. *Spore print:* not observed.

**Figure 4:**
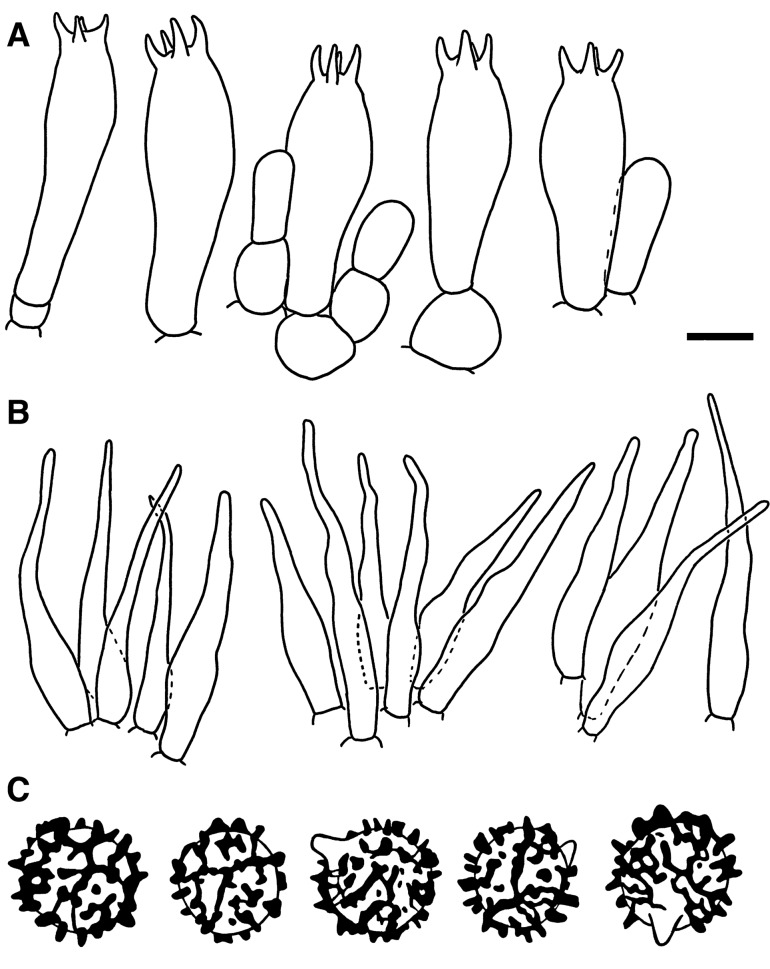
Hymenial elements of *Russula bellissima* (holotype, FH 12-127). (**A**) Basidia and basidiolae. (**B**) Marginal cells. (**C**) Spores as seen in Melzer’s reagent. Scale bar = 10 µm, but only 5 µm for spores.

**Figure 5:**
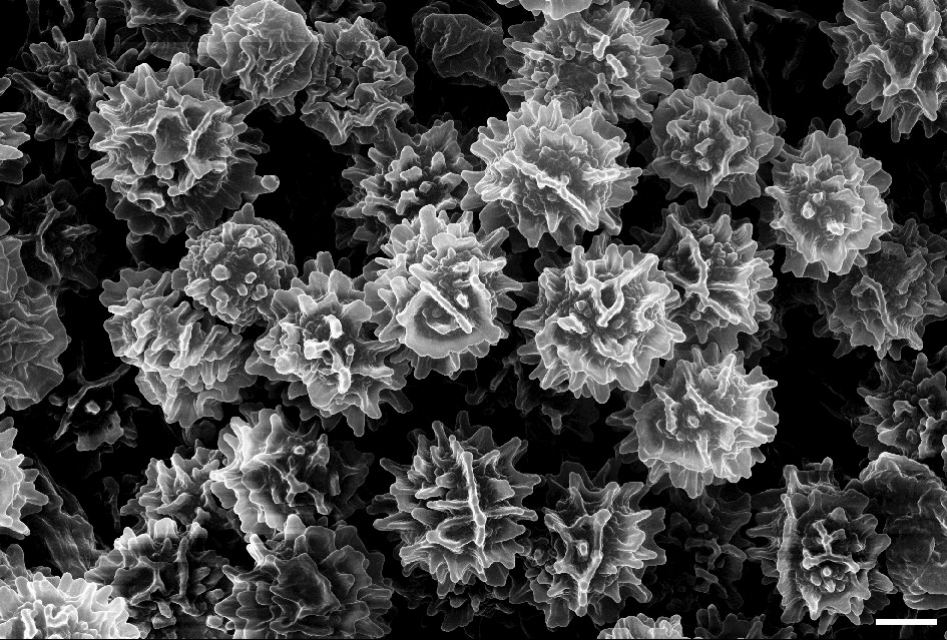
Scanning electron microscope photo of spore ornamentation. *Russula bellissima* (holotype, FH 12-127). Scale bar = 2 μm.

**Figure 6:**
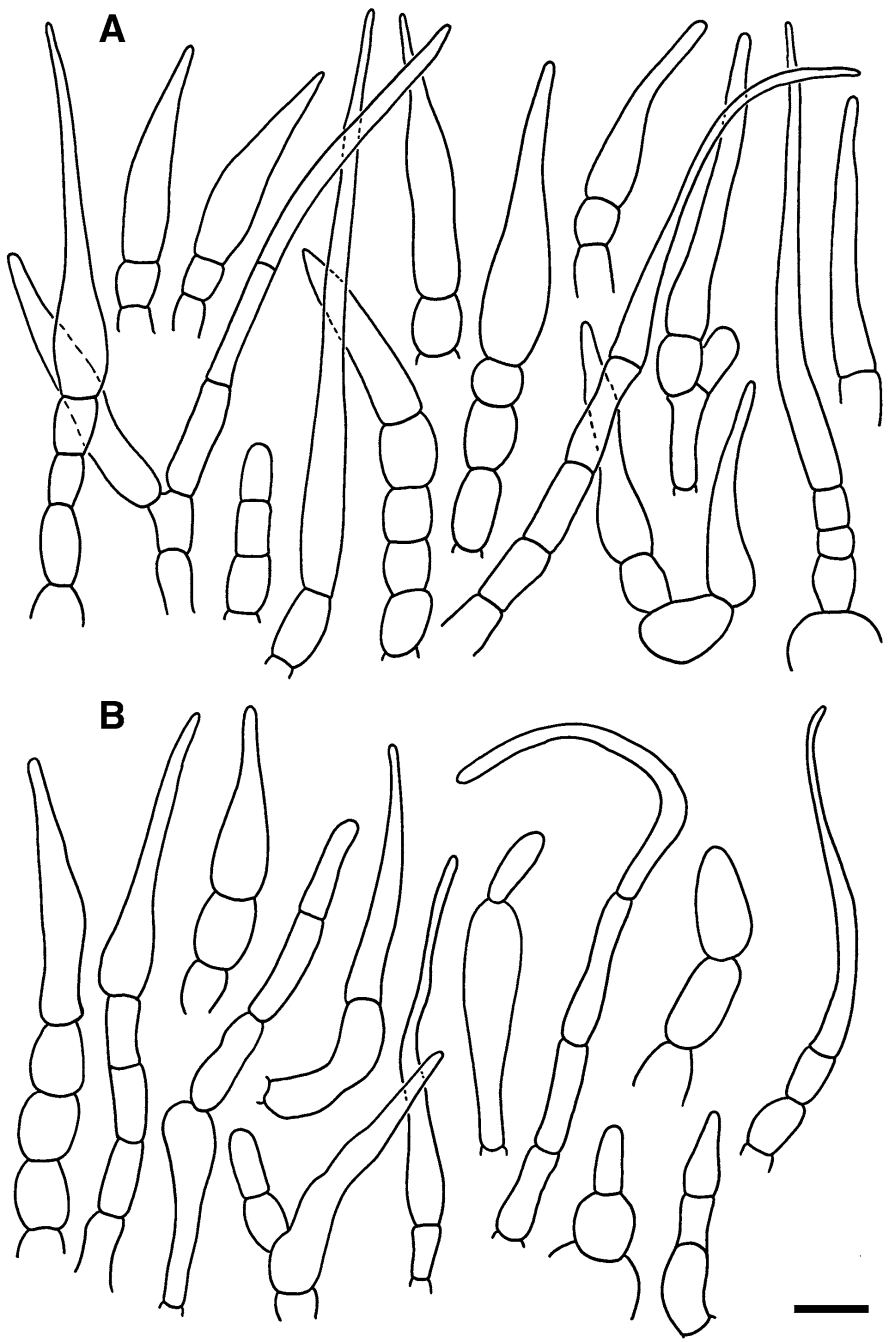
Elements of the pileipellis of *Russula bellissima* (holotype, FH 12-127). (**A**) Hyphal terminations near the pileus margin. (**B**) Hyphal terminations near the pileus centre. Scale bar = 10 μm.

*Spores* (Figs. 4, 5) (6.9–)7.3–7.8–8.3(–8.9) × (6.1–)6.8–7.2–7.6(–8.4) µm, subglobose to broadly ellipsoid, Q = 1.01–1.1–1.2(–1.29); ornamentation of moderately distant [(4–)5–6(–7) in a 3 µm diam. circle] amyloid spines or warts, (1.1–)1.2–1.4–1.6(–1.7) µm high, fused or connected by fine line connections into often long crests or wings, [(0–)1–3(–4) fusions and the same number of line connections in a 3 µm diam. circle], crests and wings frequently branched and occasionally form closed loops, isolated elements dispersed, edge of crests and wings irregularly wavy; suprahilar spot moderately large, inamyloid. *Basidia:* (30.5–)34.5–44.1–53.5(–65.0) × (10.5–)11.5–12.6–14.0(–16.0) µm, broadly clavate or obpyriform, 4-spored; basidiola cylindrical, ellipsoid or broadly clavate, ca. 5–10 µm wide. *Hymenial cystidia on lamellae sides:* absent. *Lamellae edges*: covered by densely arranged or fasciculate marginal cells. *Marginal cells:* (27.0–)38.5–46.4–54.5(–61.0) × (5.0–)5.5–6.7–7.5(–9.0) µm; subulate or narrowly lageniform, apically attenuated and constricted to ca. 1–2 µm, sometimes slightly moniliform or flexuous. *Pileipellis:* (Fig. 6) orthochromatic in Cresyl Blue, gradually passing to the underlying context, 200–300 µm deep; suprapellis 60–130 µm deep, composed of erect or ascending hyphal terminations forming a dry trichoderm, well delimited from 140 to 210 µm deep subpellis composed of horizontally oriented, strongly gelatinized narrow hyphae. Subpellis not well delimited from the underlying context, elongate hyphae gradually changing to sphaerocytes. *Acid- resistant incrustations:* absent. *Hyphal terminations near the pileus margin:* composed of long apically attenuated terminal cell and a chain of 1–4 ovoid to barrel shaped, short unbranched cells with one distinctly longer apical cell; constricted on septa, usually not flexuous, oriented towards the pileus surface, usually thin-walled, sometimes slightly thick-walled (up to 1 µm thick); terminal cells mainly subulate or lageniform, apically attenuated and acute, measuring (19–)27.5–38.3–49.0(–66.5) × (3.3–)4.5–5.8–7.0(–9.0) µm, rarely with a forked apex, mixed with dispersed, cylindrical or ellipsoid, distinctly shorter, obtuse terminal cells measuring (7.5–)11.5–17.8–29.5(–42.5) × (3.0–)4.0–4.5–5.0 µm; subterminal cells measuring (4.5–)5.5–8.3–11.5(–16.0) × 4.5–5.3–6.0(–7.0) µm. *Hyphal terminations near the pileus centre*: similar in shape and also with a mixture of long acute and short obtuse terminal cells, acute ones measuring (12.0–)22.0–35.2–48.5(–79.0) × (2.5–)3.5–4.9–6.5(–8.0) µm, obtuse ones more frequent, measuring (6.5–)8.5–12.0–15.5(–22.0) × (3.5–)4.0–4.9–6.0(–7.5) µm. *Primordial hyphae or pileocystidia*: absent. *Cystidioid hyphae and oleipherous hyphae* not observed.

*Additional material studied* THAILAND, Chiang Mai Province, Mae On District, about 3 km from Tharnthong lodges, 18° 51′ 55″ N 99° 17′ 23″ E, alt. 725 m, Dipterocarpaceae dominated forest with the presence of some *Castanopsis* trees, in small groups on loamy soil, 17 July 2012, F. Hampe (GENT FH 12-158, duplicate: MFLU12-0648).

*Note Russula bellissima* is a small species with a bright red pileus and pink colour on the stipe. This colour is distinctive and resembles North American *R. mariae*, Indian *R. intervenosa* and Asian *R. bella*. It is very unlikely that the distribution of any European or North American species is overlapping with the Thai species. However, little is known about the distributional ranges and the ecological niches of other Asian *Russula* species. Therefore discussing the morphological distinguishing characters between Asian species and *R. bellissima* is more relevant. *Russula bellissima* is not closely related to *R. bella* and it differs from this species by larger spores with a more prominent spore ornamentation, absence of hymenial cystidia on lamellae sides, and subterminally short, ellipsoid cells in the suprapellis arranged in unbranched chains of up to four^7^. The Thai species resembles and is closely related to the Indian *R. intervenosa,* but it has a more prominent spore ornamentation, hymenial cystidia (on lamellae sides) are absent, and hyphal terminations in the pileipellis are wider^22^.

#### *Russula luteonana* M. Pobkwamsuk & K. Wisitrassameewong sp. nov.

##### *Mycobank*: MB 840550

*Holotype:* THAILAND, Amnat Charoen province, Hua Taphan district, Junction near Watbochaneng , dry dipterocarp forest, alt. 145 m, 15° 41′ 28″ N 104° 31′ 41″ E, 13 July 2016, Thitiya Boonpratuang, Rattaket Choeyklin, Prapapan Sawhasan, Maneerat Pobkwamsuk, Pattrachai Juthamas, Nattawut Wiriyathanawudhiwong, Patcharee Patangwesa (BBH41120).

*Etymology* ‘Luteolus’ = yellow colour, ‘Nanus’ = small. Refer to pileus color and size of the species.

*Diagnosis* Pileus medium-sized, dry, usually yellow, spores with subreticulate amyloid ornamentation and inamyloid suprahilar spot, hymenial cystidia on lamellae sides large, lamellae edges with combination of subulate, clavate and pyriform marginal cells.

**Figure 7:**
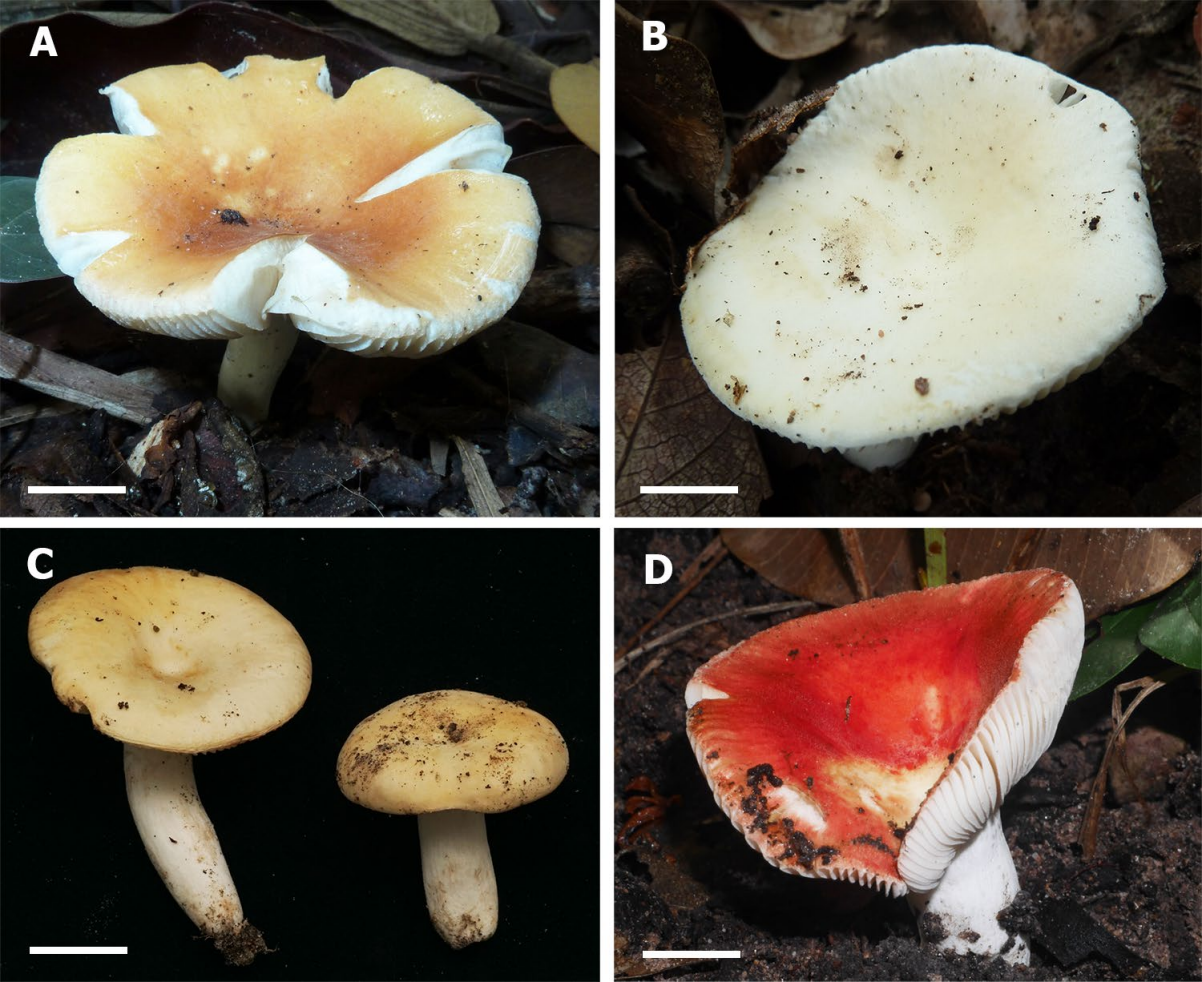
Basidiomata of *Russula luteonana*. (**A**) BBH41120 (Holotype). (**B**) BBH41121. (**C**) BBH41122. (**D**) BBH42510. Scale bar = 1 cm. Photos by Thitiya Boonpratuang.

*Pileus* (Fig. 7) medium-sized, 28‒53 mm diam., plano-convex with depressed centre, infundibuliform when mature; margin striated and radially cracking in dry condition; cuticle dry, peeling to almost ½ of radius, smooth to minutely wrinkled, dull in dry condition, color very variable, some collections pale cream and with darker pale brownish-yellow centre, other yellow brownish and with darker orange-brown centre, sometimes also bright red-brown and with discolored centre, always with rusty-brown spots especially when near the centre. *Lamellae*: 3‒5 mm deep, moderately distant, intervenose, forking near the stipe, white to cream, edges even, concolorous. *Stipe*: 26‒40 × 6‒9 mm, cylindrical or narrowed at the base, surface dry, longitudinally wrinkled, white, turning brown when bruised. *Context*: 2‒4 mm in at the half pileus radius, soft, solid, becoming partially hollow when mature, white, unchanging when cut. Taste mild; odour rather strong, fishy. *Spore print*: not observed.

**Figure 8:**
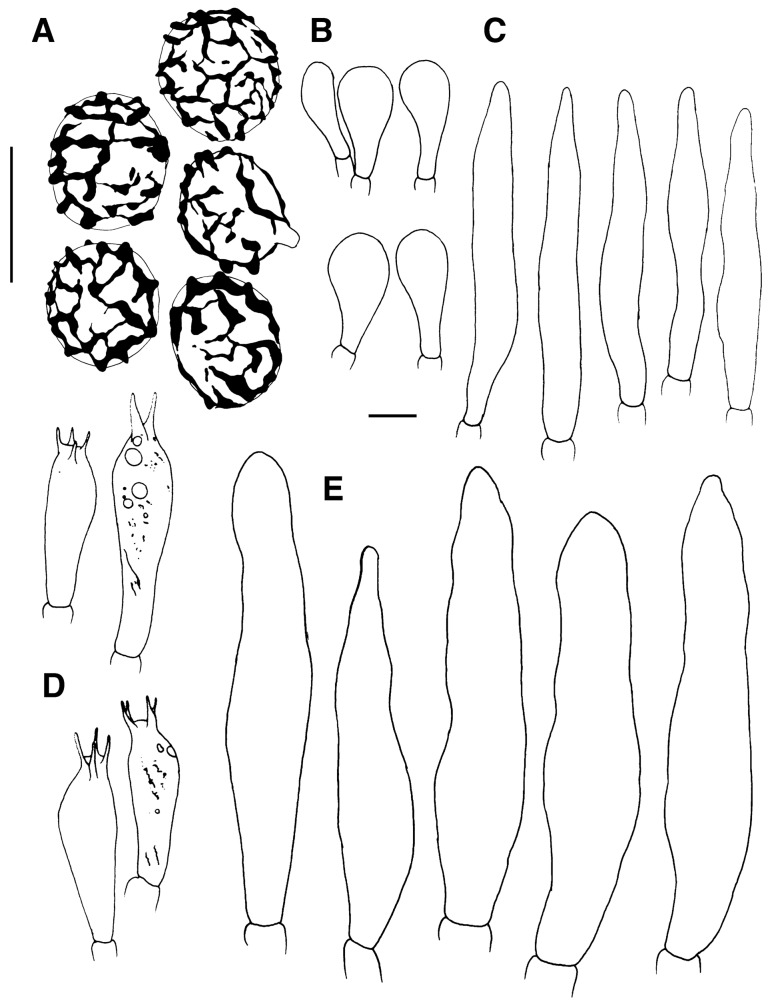
Hymenial elements of *Russula luteonana* (holotype, BBH41120). (**A**) Spores as seen in Melzer’s reagent. (**B**) Clavate marginal cells. (**C**) Subulate marginal cells. (**D**) Basidia. (**E**) Hymenial cystidia on lamellae sides. Scale bar = 10 µm.

**Figure 9:**
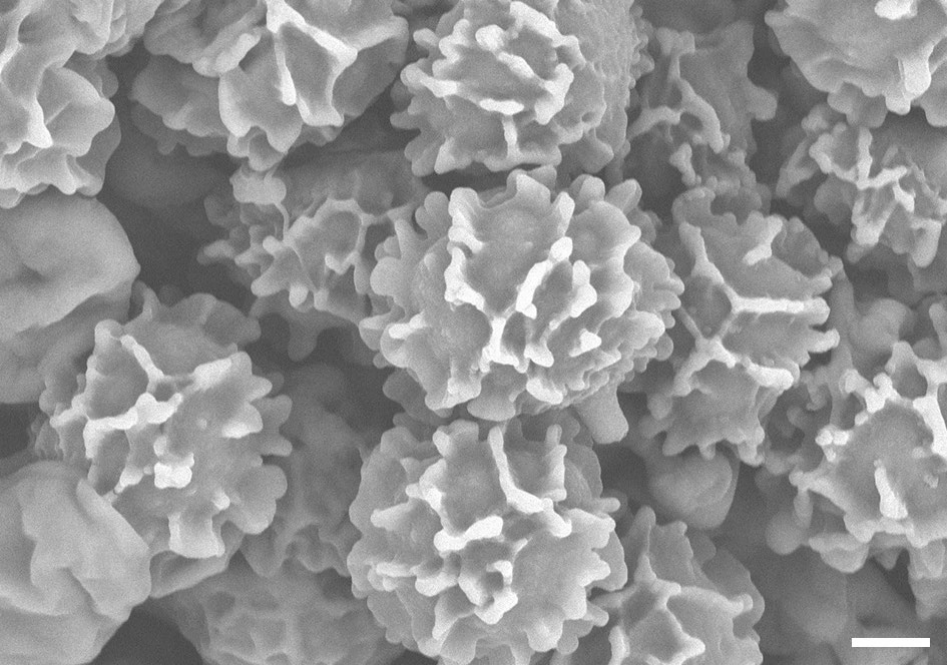
Spore ornamentation taken from scanning electron microscope. *Russula luteonana* (holotype, BBH41120). Scale bar = 2 μm.

**Figure 10:**
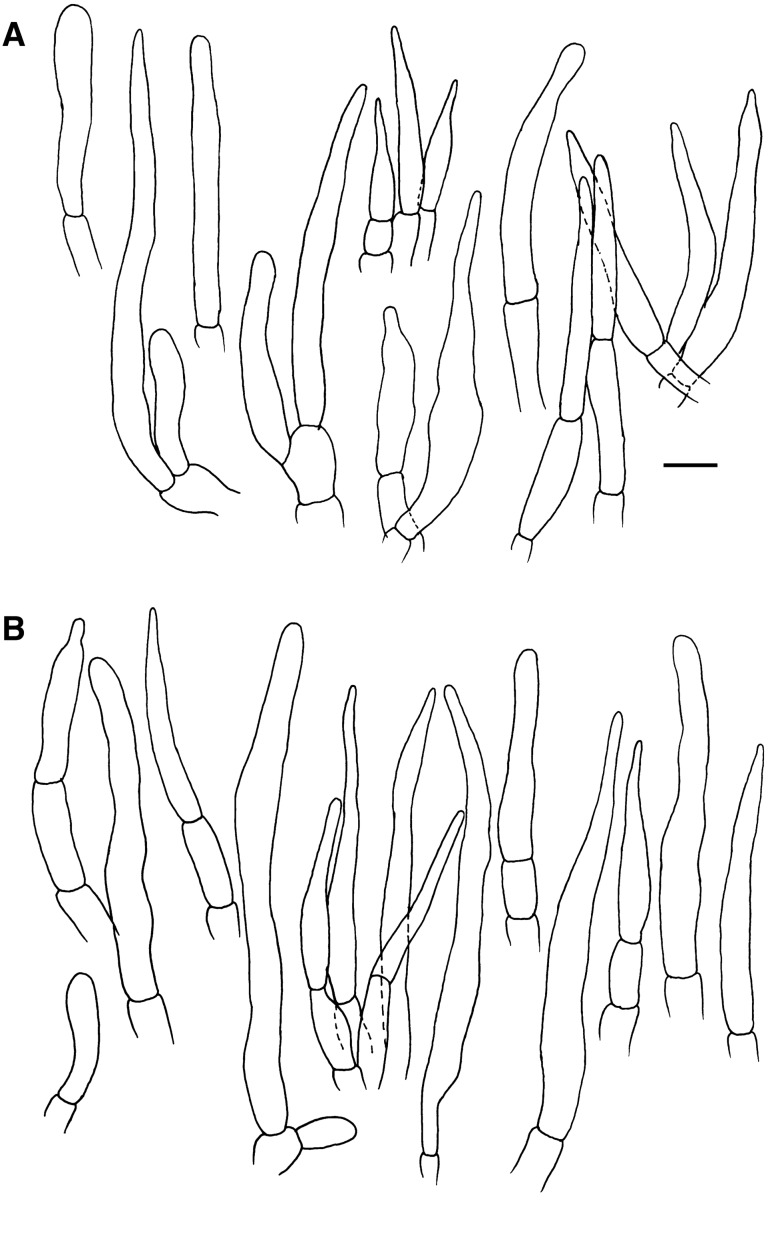
Elements of the pileipellis of *Russula luteonana* (holotype, BBH41120). (**A**) Hyphal terminations near the pileus margin. (**B**) Hyphal terminations near the pileus centre. Scale bar = 10 μm.

*Spores* (Figs. 8, 9) (7.4‒)8.1‒8.6‒9(‒10.1) × (6.1‒)7.4‒7.5‒7.9(‒9.1) μm, subglobose to broadly ellipsoid, Q = (1.03‒)1.09‒1.15‒1.20(‒1.30), ornamentation of moderately distant, obtuse, (0.7‒)1.1‒1.3‒1.5(‒1.9) μm high spines, connected by abundant line connections [(0‒)3‒6(‒8) in in a 3 µm diam. circle], branched, forming an incomplete reticulum, crest irregularly wavy and occasionally fused [(0‒)1‒2(‒5) fusions in the circle], isolated elements rare; suprahilar spot inamyloid. *Basidia*: (29‒)34.5‒39.1‒44(‒51.5) × (10‒)12‒13.2‒14.5(‒16.5) μm, clavate, 4-spored, rarely 2-spored, basidiola subcylindrical to subclavate, (25.5‒)30‒35.4‒41(‒47) × (9‒)11‒12.2‒14 (‒16) μm. *Hymenial cystidia on lamellae sides:* usually protruding over other elements of hymenium, widely dispersed (< 350/mm^2^), (65‒)78‒92.1‒106.5(‒132) × (10.5‒)14‒17.4‒21(‒24) μm, fusiform to lageniform, apically obtuse to subacute, thin- or slightly thick-walled; contents homogenous, optically empty, negative in sulfovanillin. *Lamellar edges:* with dispersed basidia; *Marginal cells*: very abundant, mainly long and apically acute, resembling terminal cells in the pileipellis, (28.5‒)41.5‒55‒69(‒93) × (5.5‒)7.5‒9.0‒10.5(‒13) μm, fusiform, rarely lanceolate or lageniform, often fasciculate; mixed with less frequent, distinctly shorter, broadly clavate or obpyriform elements μm, (12.4‒)20.1‒25.7‒31.2(‒44.0) × (5.2‒)‒8.9‒10.9‒12.8(‒14.8) μm. *Pileipellis*: (Fig. 10) orthochromatic in Cresyl blue, sharply delimited from the underlying context, 110‒350 um deep; suprapellis a trichoderm of ascending or erect hyphal terminations, non-gelatinized, subpellis composed of dense, strongly gelatinized, horizontally oriented, narrow hyphae. *Acid-resistant incrustations*: absent. *Hyphal terminations near the pileus margin*: mainly unbranched, apically often flexuous, usually composed of distinctly longer terminal cells and a single subterminal short cells, thin-walled; terminal cells of two distinct types, either long and apically attenuated or short, subcylindrical and obtuse, constricted on septa, the long type (27.5‒)44.5‒60.7‒76.5(‒100.5) × (4.0‒)5‒6.6‒8(‒11.3) μm, subulate, narrowly fusiform or narrowly lageniform, apically acute ; the short type (16.4‒)24.7‒36.2‒47.8(‒71.9) × (3.7‒)4.7‒5.7‒6.8(‒7.7) μm, cylindrical, rarely narrowly clavate or somewhat apically narrowed, occasionally moniliform; subterminal cells shorter, mainly unbranched, 5‒8 μm wide. *Hyphal terminations near the pileus centre:* similarly with terminal elements of two types, the longer type (20‒)37.5‒53‒69(‒90) × (3.5‒)5‒6.1‒7.5(‒9) μm, subulate and acute or subcylindrical and obtuse, the shorter type (12‒)18.5‒31.4‒44.5(‒65.5) × (3‒)4‒5.0‒6(‒7) μm, subcylindrical, cylindrical, rarely narrowly clavate. *Primordial hyphae or pileocystidia:* absent. *Cystidioid hyphae and oleipherous hyphae:* in subpellis absent.

*Additional material studied* THAILAND, Amnat Charoen province, Hua Taphan district, Junction near Watbochaneng, dry dipterocarp forest, 13 July 2016, 3 collections from different mycelia at this site, Thitiya Boonpratuang, Rattaket Choeyklin, Prapapan Sawhasan, Maneerat Pobkwamsuk, Pattrachai Juthamas, Nattawut Wiriyathanawudhiwong, Patcharee Patangwesa, (BBH41121, BBH41122, BBH41125); ibid., 29 May 2017, Thitiya Boonpratuang, Rattaket Choeyklin, Maneerat Pobkwamsuk, Nattawut Wiriyathanawudhiwong, Tuksaporn Thummarukcharoen (BBH42510).

*Note Russula luteonana* is extremely variable but based on the pileus colour we can eliminate some species with purple or green tints. If we combine this with the white or nearly so stipe, it can only be confused with either *R. bella* (if it has redder colouration) or *R. orientipurpurea*. The unique character of *R. luteonana* is the large cystidia which range 14–21 μm in width and are often also obtuse. While *R. bella* has many microscopic characters that distinguish it from this proposed species (e.g. smaller spores, narrower hymenial cystidia), *R. orientipurpurea* resembles, in many aspects, the Thai species (i.e. relatively large spores, obtuse and relatively wide hymenial cystidia on the lamellae sides, and usually only one unbranched short cell below the terminal cell of hyphae in pileipellis). Distinguishing features of these two species are the more prominent spore ornamentations and the often acute hymenial cystidia of *R. luteonana*^7^*.*

## Discussion

Members of *Russula* subsect. *Amoeninae* were identified in the field based on the typical dryness of the pileus, the velvety-granulose aspect of pileus cuticle, and white lamellae. Later each collection was checked under the microscope for the absence of cystidia in the pileipellis. In the field, both new species are distinct from many other species, because of the pileus with red, yellow or brown tints and completely without purple or greenish colours. The stipes of both species are white or only partly flushed by pink, and never with purple or deep red on more than half of their surface. There is a high degree of infraspecific variability of pileus colours in *Amoeninae* and therefore field observations need sufficient sampling or further verifications under the microscope to identify them accurately to species^7,23^.

Microscopically, all *Amoeninae* representatives have a distinctive spore ornamentation composed of relatively prominent ridges and crests connected also by line connections to form a subreticulate to reticulate ornamentation. Both new species, *R. bellissima* and *R. luteonana*, typically have relatively large spores with prominent spore ornamentations that distinguishes them clearly from many of the Asian *Amoeninae* species (Table 1). Important features for species delimitation within *Amoeninae* are the hymenial cystidia and hyphal terminations in the pileipellis. While hymenial cystidia on lamellae sides are completely absent in *R. bellissima*, they are very large and often base-inflated (lageniform or subulate) in *R. luteonana*. Thus these microscopic structures distinguish both species from all known Asian *Amoeninae*. Hyphal terminations in the pileipellis are very different between the two newly described species and they prove importance of specific characters used already for European species, e.g. size of subterminal cells and their number^7,23^. *Russula bellissima* has relatively short terminal cells measuring up to 50 µm and usually 2–3 short, subglobose subterminal cells that make it similar to *R. intervenosa* from India^24^. *Russula luteonana* has terminal cells usually longer than 50 µm and zero to one cylindrical subterminal cells which make it similar to *R. orientipurpurea*^7^.

**Table 1:** Comparison of selected morphological characters of known *Amoeninae* species from Asia and species described in this study.

Pileus size (mm)	*R. bella* (1)	*R. orientipurpurea* (1)	*R.* sp. (1)	*R. intervenosa* (2)	*R. pseudoamoenicolor* (3)	*R. pauriensis* (4)	*R. mukteswarica* (5)	*R. bellissima* (6)	*R. luteonana* (6)
	20–50	52–60	60	26–49	50–100	53–63	65–130	10–50	28–53
**Pileus colour**									
Bright red	1			1	1			1	1
Pink	1			1					
Grey		1	1			1			
Brown									1
Purple		1			1		1		
Violet			1		1	1	1		
Green						1	1		
Bright yellow				1			1		1
Cream or pale yellow		1							1
**Stipe colour**									
Almost white	1	1	1						1
Partly pink				1	1		1	1	
Partly purple				1	1	1	1		
Partly violet						1			
**Spore size**									
Length (µm)	6.5–7.7	6.9–7.8	6.5–7	7–8	6–9.5	6–8	7.6–9.3	7.3–8.3	8.1–9.0
Width (µm)	5.3–6.0	6–6.9	5.6–6.2	6.5–7	5–8	5.5–7	7.3–8.2	6.8–7.6	7.4–7.9
**Spore ornamentation**									
Height (µm)	0.4–1.0	0.6–1	0.7–1.2	0.6–0.9	Up to 2	Up to 2	0.75	1.2–1.6	1.1–1.2
Subreticulate	1	1	1	1	1	1	1	1	1
Reticulate		1							
**Hymenial cystidia**									
Length (µm)	52–75	74.5–101	12.5–16.5	29–34	90–117	55–135	80–110		78–106.5
Width (µm)	7.5–10.5	10.5–15		10–12.5	10–21	12–22	11–17		14–21
Cylindrical or clavate	1								
Subulate or lageniform			1						1
Fusiform		1	1	1	1	1	1		1
Obtuse	1	1			1	1			1
Acute			1	1		1			
**Marginal cell**									
Length (µm)	38.5–63	48–88	42.5–56.5	32–39	30–85	36–68	70–100	38.5–54.5	41.5–69
Width (µm)	5.5–7.5	5.5–10.5	5.5–7	5.5–7	7–10	8–15	11–17	5.5–7.5	7.5–10.5
Different from sides	1	1	1	1				1	1
**TC margin**									
Length (µm)	47–76	55.5–89	60–85	39–47	11–65	9–64		27.5–49	24.7–76.5
Width (µm)	5–7	5–7	4.5–6	2.5–4.5	4–10	4–10	5–11	4.5–7	4.7–8
**Subterminal cell**									
Number	1–2	0–2	1–2			2–4	2–4	1–4	0–1
Width (µm)					Up to 14	Up to 12		4.5–6	4–8
**TC centre**									
Width (µm)	4.5–7.5	4.5–7.5	3–4					3.5–6.5	4–7.5

Sources of descriptions: (1) Wisitrassameewong et al.^7^, (2) Crous et al.^22^, (3) Hyde et al.^25^, (4) Das et al.^24^, (5) Das et al.^26^, (6) this study.

The first and only phylogenetically confirmed records of *Amoeninae* members in Thailand were published by Wisitrassameewong et al.^7^, and they were represented by two samples with GenBank accession numbers AB459514 and AB854679. In our ITS analysis both these samples cluster within the *R. bellisima* clade together with three other samples from Thailand, Laos and Singapore. Our ITS tree covers nearly all previously published species of *Amoeninae* in Asia and all available sequence data of this group from public databases. The only Asian species without available DNA that are not included in our analyses are *R. mukteswarica* K. Das, S.L. Mill., J.R. Sharma & R.P. Bhatt and *R. punicea* W.F. Chiu. Both of these species are morphologically very different from species described here. *Russula mukteswarica* has a purple and green coloured pileus and low spore ornamentation (only 0.75 μm), and the *R. punicea* has small spores (up to 7 μm) and small hymenial cystidia^7^. In conclusion, our study does not confirm any species of *Amoeninae* previously described or recorded from India, Korea, Japan or China occurring in Thailand. We were unable to locate any sequence record for *R. luteonana* in public sequence databases, but *R. bellisima* is represented by two additional sequences from Thailand and two more from Laos and Singapore. The Thailand sequences originated from a dry dipterocarp forest (GenBank accession number AB854679) and from an evergreen dipterocarp forest with annual precipitation of approximately 1030 mms (GenBank accession number AB459514)^27,28^. The Laos record (UNITE accession number UDB033872) is from an urban area of Vientiane city on the northeastern Thai border and the Singapore record (GenBank accession number MZ519838) is also from Singapore Botanical Garden collected with *Shorea leprosula*. Because all collections of our new species are located in Mainland Southeast Asia, this result suggests that this area’s endemic *Russula* diversity developed under specific climate, geomorphology and available ECM host range, such as already suggested for southwestern Himalayas^29^. In this respect it is worth to mention that sister to both our new species collected in dry dipterocarp forests are sequestrate species (Figs. 1, 2)^30^ and both species can be members of two independent seasonal drought tolerant lineages within *Amoeninae*. Despite our effort, we did not collect any sequestrate species of *Russula* from dry dipterocarp forests, but during our field excursions, members of our expedition collected sequestrate *Lactarius* and *Entoloma*^31,32^.

The fungal flora of dipterocarp forests is still very poorly known, but there is a multitude of evidence that roots of Dipterocarpaceae trees are colonized by ectomycorrhizal fungi^33^. Based on sequencing of root tips, Dell et al.^4^ estimated that the fungal richness of dry dipterocarp forests in Thailand is comparable to other tropical rain forest sites, but the phylogenetic community structure has elements of both tropical and temperate ecosystems^4^. They confirmed insufficient knowledge of fungal species diversity of this habitat, only 9 of the 69 species matched with sequences from public databases at the 97% sequence similarity cut-off and only four of these taxa were identified to species based on the available reference sequences and identifications. In terms of species richness, Russulaceae lineage was the richest and a *Russula* cf. *pectinata* was the most frequent molecular operational taxonomic unit retrieved from all dataset. Phosri et al.^3^ reported that the diversity of ECM fungi in dipterocarp forests in Northern Thailand is the second most abundant after Fagaceae forest and the *Russula-Lactarius* lineage was among the dominant fungal groups. In lowland dry dipterocarp forest of Malaysia, *Russula* is reported as one the most abundant genera found from bulk soil and root tip samples^34^. Recent extensive mycological surveys in Southeast Asia resulted in the discoveries of several new ECM species specific to Dipterocarpaceae dominated forests, for example species of *Lactarius* subg. *Plinthogalus* (Burl.) Hesler & A.H. Sm. From Malaysia^35,36^, *Amanita* Pers. and *Lactifluus* (Pers.) Roussel from northern Thailand^37–40^, *Erythrophylloporus* Ming Zhang & T.H. Li from Northern Thailand^41^ and *Sutorius* Halling, Nuhn & Fechner from northern and northeastern Thailand^42^. New *Amoeninae* species are not the first *Russula* members described from dry dipterocarp forests, also *R. aurantiopectinata* was collected and is reported with *Dipterocarpus tuberculatus* from this type of habitat^12^. However, our study is the first discussing possibly unique ectomycorrhizal communities and species adapted to this kind of ecological and climatic conditions.

To understand distributional ranges of *Russula* species collected in dry dipterocarp forests, we need to understand their niche limits in term of habitat specificity, ecological adaptations to certain climate and soil conditions, and their life strategy in general. Based on sequencing of Dipterocarpaceae root samples, Sato et al.^43^ suggested that specific lineages of closely related dipterocarp taxa are associated with some specific ECM forming Basidiomycota OTUs and have uniquely characteristic community structure. However, seedling experiments of Dipterocarpaceae species with contrasting soil specializations proved that there is little host specificity and soil environment was the primary determinant of ectomycorrhizal diversity^44^. This result is in contrast with the study of Essene et al.^34^, reporting that majority of the taxa detected in root tip samples had not only restricted preference of soil type (either sand or clay soil), but also were associated with only one species of *Shorea*. Two different *Shorea* tree species on the same soil type were colonized by different ECM fungal communities. These results suggested that these biotic and abiotic factors might have played a role in structuring ECM fungal communities in Dipterocarpaceae forests.

In Thailand, wild edible mushrooms are one of the three main non-timber forest products collected during the rainy season^45^. Mushroom foraging is considered to be a recreational activity, seasonal food source, or an option for additional household income. Several species of *Russula* are considered choice for consumption in rural regions, particularly in northern and northeastern Thailand and they are a commonly eaten mushrooms in the region due to their high yields in Thai forests. Various morphological types of *Russula* are frequently sold in local markets e.g. reddish cap (called *R.* cf. *emetica* or *R.* cf. *rosacea*), whitish cracked cap (called *R.* cf. *alboareolata*), cream to brownish cap (called *R.* cf. *heterophylla*) or greenish cap (called *R. virescens*)^8,46^. Due to the pileus colour, *R. bellissima* and *R. luteonana* probably can match *R.* cf. *rosacea* and *R.* cf. *heterophylla* morphotypes, respectively. Accordingly, two of *R. bellissima* ITS sequences retrieved from GenBank were labeled as *R.* cf. *rosacea*. Globally, two *Amoeninae* members, *R. mariae* and *R. violeipes* are known as edible fungi used for consumption as well as members of other related lineages like subsections *Virescentinae* and *Heterophyllae* (Fr.) Jul. Schäff. that are well known edible fungi^47^. Therefore, it is likely that the two species described in this study are edible. However, the correct identification of well documented edible species is important because a number of *Russula* species are reported as poisonous^48,49^ or can cause gastrointestinal irritations, and the latter ones include *Russula* collections identified as *R.* cf. *rosacea*^50^.

## Materials and methods

### Studied collections and morphological study

Our collections originated from dry dipterocarp forests in two distant areas of Thailand collected in 2012–2017. Two collections (FH12-127 and FH12-158) were collected at two locations in northern Thailand at altitudes of 500–700 m*.* These samples were deposited in Mae Fah Luang University (MFLU) and Ghent University (GENT). Five collections (BBH41120, BBH41121, BBH41122, BBH41125, BBH42510) were collected in northeastern Thailand, at altitudes of 100–200 m. These five collections were deposited in BIOTEC Bangkok Herbarium of National Biobank of Thailand (BBH). Macroscopic characters were all recorded based on fresh material. For the terminology of macroscopic features, we followed Vellinga et al.^51^. The colour codes follow Kornerup and Wanscher^52^.

To describe all microscopic features, we used the description template and terminology of Adamčík et al.^6^. Microscopic characters were studied on dried material mainly in Congo Red^53^, except for spore morphology, which was observed in Melzer reagent. Chemical tests were applied with Cresyl Blue^54^, sulfovanillin^55^, and carbolfuchsin^56^ to observe colour changes, incrustations, and cystidia contents. Line drawings and measurements of all microscopic characters were done using Nikon Eclipse Ni (Nikon Instrument Inc., Japan), with the aid of the software NIS-element BR 5.02.03 at a projection scale of 2000 ×. Scanning electron microscopy (SEM) images were done using Zeiss Auriga crossbeam microscope at a 6330 × magnification and FESEM Hitachi SU5000 at a 5000 × magnification. Ranges of measurements were estimated as average plus/minus two times of standard deviation; in parenthesis are minimum and maximum values. Q value corresponds to length/width ratio of spores. The central value in italics represents an average. The measurements of all microscopic characters of a species were observed for three collections if available with 30 measurements per collection.

### DNA extraction, PCR amplification and sequencing

Genomic DNA was extracted from dried materials using E.Z.N.A. Forensic DNA kit (Omega Bio-Tek). The amplification of the internal transcribed spacer region (ITS) of the nuclear ribosomal DNA was done using primers ITS1-F^57^ and ITS4^58^. The primers RPB2-6F and fRPB2-7cr^59^ and the primers MS1 and MS2^58^ were used to amplify the second largest subunit of RNA polymerase II (*rpb2*) and mitochondrial small subunit ribosomal DNA region (mtSSU), respectively. DNA sequencing of the successful PCR products using the same primers was performed by an ABI 3500 Genetic analyzer (Thermo Fisher Scientific) at the National Biobank of Thailand. Obtained sequences were checked and edited using FinchTV 1.4 (Geospiza, Inc.) and then assembled in MEGA X^60^.

### Alignment and phylogenetic analyses

Phylogenetic analysis was based on three DNA regions: ITS nrDNA, mtSSU and *rpb2*. The DNA sequences of *Amoeninae* members used for the multi-loci analysis were downloaded from GenBank (https://www.ncbi.nlm.nih.gov/genbank/) based on the previous study of Wisitrassameewong et al.^7^ (Supplementary Table 1). Three other members of *R.* subg. *Heterophyllidiae, R.* aff. *crustosa*, *R. grisea*, and *R. vesca*, were chosen as an outgroup for this multi-locus analysis. For better estimation of overall diversity of *Amoeninae*, we downloaded available ITS *Amoeninae* sequences with special emphasis to Asian taxa. Our selection of ITS sequences covers species recognized by Wisitrassameewong et al.^7^ and additionally also all Asian members with at least 90% BLAST similarity to any described *Amoeninae* member. We also searched the UNITE database (https://unite.ut.ee/) for Asian samples matching *Amoeninae* with a 3% identity threshold. Based on previous phylogenetic studies, members of the other subsections of *R.* subg. *Heterophyllidia* were included in the ITS dataset and members of *R.* subsect. *Virescentinae* Singer was selected as the outgroup. All datasets were aligned using MAFFT^61^. We inferred phylogenies for each single-gene dataset using Maximum Likelihood (ML). ML analyses were performed using RAxML 8.2.12^62^. The parameters for RAxML analyses were the GTRGAMMA model and the rapid bootstrapping algorithm for 1,000 replicates. We compared the tree topology between different single-gene trees and examined for conflict at nodes with bootstrap support value (BS) above 70% and posterior probability (PP) higher than 0.90. A conflict can be considered as significant if two different relationships for the same set of taxa were observed among different single-gene phylograms. A concatenated dataset of ITS + *rpb2* + mtSSU was constructed using BioEdit 7.2.5^63^. jModelTest 2.1.6 was used to estimate the best-fit model of substitution for each partition^64^. The models based on Bayesian information criterion (BIC) were selected as follows: K80 + G for *rpb2*pos1, *rpb2*pos2 and *rpb2*pos3, TIM1 + G for mtSSU, K80 + G for ITS1, TPM1 for 5.8 s rDNA and TrNef + G for ITS2 regions. Bayesian Inference (BI) analysis was executed using MrBayes 3.2.7a^65^. The analysis was executed for four independent runs with four chains each that was run for 10 million generations and sampled every 100th tree until the standard deviation of split frequency was less than 0.01. Before finalizing a consensus tree, the convergence and ESS values were checked using Tracer 1.6^66^. The burn-in of 10,000 for each run was used. All phylogenetic analyses were done using the CIPRES Science Gateway^67^.

## Supplementary Information

Supplementary Table 1.
