# Supplementary material for: Two new Russula species (fungi) from dry dipterocarp forest in Thailand suggest niche specialization to this habitat type
Source: Sci Rep. 2022 Feb 18;12:2826. doi: 10.1038/s41598-022-06836-x (PMC8857229; doi:10.1038/s41598-022-06836-x)
Supplement: Supplementary file 1 — Supplementary Table 1. [file 41598_2022_6836_MOESM1_ESM.docx]

**Supplementary table 1.** Specimens and GenBank accession number of DNA sequences used in the phylogenetic analyses. The newly generated sequences in this study are presented in boldface. The A is indicated for angiocarpic specimens.

| Species | Voucher collection | Locality | Accession number | | |
| --- | --- | --- | --- | --- | --- |
|  |  |  | nrITS | *rbp2* | mtSSU |
| *R. aeruginea* | AT2003017 |  | DQ421999 | – | – |
| *R.* aff. *crustosa* | 31/BB 06.616 | Canada | – | KU237747 | KU237305 |
| *R.* aff. *mariae* | SAV F–4484 | USA | MT017557 | – | MT417192 |
| *R.* aff. *mariae* | SAV F–4493 | USA | MT017558 | – | MT417193 |
| *R.* aff. *mariae* | SAV F–4564 | USA | MT017559 | – | MT417194 |
| *R.* aff. *mariae* | L2Z_4_11 | USA | JX030253 | – | – |
| *R. andaluciana* (A) | BM360 (Type) | Spain | MK105627 | – | – |
| *R. andaluciana* (A) | 39198 | Spain | MK105628 | – | – |
| *R. alachuana* | SAV F20113 | USA | MT017542 | MT417198 | MT417186 |
| *R. alachuana* | SAV F–20108 | USA | MT017543 | MT417199 | MT417187 |
| *R. alachuana* (*Russula* sp.) | FLAS-F-60871 | USA | MH016858 | – | – |
| *R. alachuana* (*Russula* sp.) | FLAS-F-61265 | USA | MH211844 | – | – |
| *R. alachuana* (*Russula* sp.) | FLAS-F-61313 | USA | MH211876 | – | – |
| *R. alachuana* (*Russula* sp.) | S.D. Russell MycoMap 6332 | USA | MK532805 | – | – |
| *R. amoena* | SAV F–1352 | Slovakia | MT017545 | MT417200 | MT417185 |
| *R. amoena* | SAV F–3147 | Slovakia | MT017544 | MT417202 | MT417190 |
| *R. amoenicolor* | 311IX76 |  | AY061655 | – | – |
| *R. amoenicolor* (*Russula* sp.) | 02DBH070700024 | France | FN610951 | – | – |
| *R. amoenicolor* (*Russula* sp.) | 02DBH070700026 | France | FN610953 | – | – |
| *R. anatina* | FH2011BT310 | Germany | MT738285 | – | – |
| *R. bella* | SFC20120722–03 | South Korea | MT017553 | MT199642 | MT196930 |
| *R. bella* | SFC20170819–05 | South Korea | MT017552 | MT199643 | MT196931 |
| *R. bella* | SFC20170819–10 | South Korea | MT017555 | MT199644 | MT196932 |
| *R. bella* | SFC20170731–02 | South Korea | MT017556 | MT199645 | MT196936 |
| *R. bellissima* sp. nov. | FH12-127 (Type) | Thailand | MZ297950 | MZ303710 | MZ297941 |
| *R. bellissima* sp. nov. | FH12-158 | Thailand | MZ297951 | – | MZ297942 |
| *R. bellissima* (*Russula* cf. *rosacea*) | CUB:Microbiology KHS5 | Thailand | AB459514 | – | – |
| *R. bellissima* (*Russula* *rosacea*) | REME | Thailand | MN580115 | – | – |
| *R. bellissima* (*Russula* sp.) | Rus1 | Thailand | AB854679 | – | – |
| *R. bellissima* (*Russula* sp.) | HNL501695 | Laos | UDB033872 | – | – |
| *R. bellissima* sp. nov. | SL1157 | Singapore | **MZ519838** | – | – |
| *R.* cf. *amoenicolor* | SAV F–20302 | Greece | MT017546 | MT417196 | MT417188 |
| *R.* cf. *amoenicolor* | SAV F–20324 | Greece | MT017547 | MT417197 | MT417189 |
| *R.* *crustosa* | BPL265 | USA | KT933966 | – | – |
| *R. faustiana* | FH2011BT010 | Germany | MT738276 | – | – |
| *R. grisea* | 449/BB 07.184 | Slovakia | – | KU237795 | KU237355 |
| *R. grisea* | SAV F-1395 | Slovakia | MT738286 | – | – |
| *R. intervenosa* | CUH AM273 (Type) | India | KT824241 | – | – |
| *R. ionochlora* | FH2010BT141 | Germany | MT738289 | – | – |
| *R. luteonana* sp. nov. | RSPG00468 (Type) | Thailand | MZ297955 | MZ303714 | MZ297946 |
| *R. luteonana* sp. nov. | RSPG00469 | Thailand | MZ297956 | MZ303715 | MZ297947 |
| *R. luteonana* sp. nov. | RSPG00470 | Thailand | MZ297954 | MZ303713 | MZ297945 |
| *R. luteonana* sp. nov. | RSPG00473 | Thailand | MZ297953 | MZ303712 | MZ297944 |
| *R. luteonana* sp. nov. | RSPG00586 | Thailand | MZ297952 | MZ303711 | MZ297943 |
| *R. mariae* | 546/BB 07.038 | USA | – | KU237824 | KU237384 |
| *R. medullata* | SAV F-1596 | Slovakia | MT738281 | – | – |
| *R. mustelina* | FH12-226 | Germany | KT934005 | – | – |
| *R. nitida* | PC BB2004-272 | USA | EU598164 | – | – |
| *R. orientipurpurea* | SFC20170819–08 | South Korea | MT017550 | MT199638 | MT196926 |
| *R. orientipurpurea* | SFC20170725–37 (Type) | South Korea | MT017548 | MT199639 | MT196927 |
| *R. orientipurpurea* | SFC20170821–22b | South Korea | MT017549 | MT199640 | MT196928 |
| *R. orientipurpurea* | SFC20170726–47 | South Korea | MT017551 | MT199641 | MT196929 |
| *R. pauriensis* | CAL:1395 (Type) | India | MF535185 | – | – |
| *R. pseudoamoenicolor* | CAL1330 (Type) | India | KX234819 | MT199646 | MT196937 |
| *R. rostraticystidia* (A) | H6165 (Type) | Australia | EU019938 | – | – |
| *R. variispora* (A) | H5855 (Type) | Australia | EU019934 | – | – |
| *R. sp.* (A) | F33_12 | Australia | KY697596 | – | – |
| *R. vesca* | 45/BB 06.525 | Mexico | – | KU237751 | KU237309 |
| *R. vesca* | BPL284 | USA | KT933978 | – | – |
| *R. violeipes* | 542/BB 07.273 | Slovakia | – | KU237820 | KU237380 |
| *R. violeipes* | 208IS76 |  | AY061726 | – | – |
| *R. violeipes* | 1792 | Italy | JF908655 | – | – |
| *R. violeipes* | MC00-501 | Denmark | UDB000001 | – | – |
| *R. violeipes* | TL-6879 | Denmark | UDB000116 | – | – |
| *R. violeipes* | O-F-249899 | Norway | UDB036092 | – | – |
| *R. virescens* | HJB9989 |  | DQ422014 | – | – |
| *R.* sp. | 180717C17 | China | MK748180 | – | – |
| *R.* sp. | 180717C9 | China | MK748181 | – | – |
| *R.* sp. | 180824H21 | China | MK748183 | – | – |
| *R.* sp. | SFC20160726–13 | South Korea | MT017561 | MT199647 | MT196938 |
| *R.* sp. | SAV F–20115 | USA | MZ519726 | MT417201 | – |
| *R.* sp. | SAV F–20117 | USA | MT017562 | MT417195 | – |
| *R.* sp. | SAV F–20134 | USA | MT017563 | – | MT417205 |
| *R.* sp. | SAV F–4063 | USA | MT017560 | MT417203 | MT417191 |
| *R.* sp. | FLAS-F-61195 | USA | MH211804 | – | – |
| *R.* sp. | G3060 | India | UDB0754995 | – | – |
| *R.* sp. | G3547 | Estonia | UDB0293621 | – | – |
